# 1,8-Cineole improves hepatic fibrosis by inhibiting HIF-1α/BNIP3/PINK1/Parkin-dependent mitophagy signaling and inducing apoptosis in activated hepatic stellate cells

**DOI:** 10.3389/fphar.2026.1873519

**Published:** 2026-09-03

**Authors:** Lifeng Qin, Jiawu Tan, Chan He, Maoyin He, Jiqiao Zhang

**Affiliations:** 1 Hubei Provincial Key Laboratory of Occurrence and Intervention of Rheumatic Diseases, Minda Hospital of Hubei Minzu University, Enshi, Hubei, China; 2 Hubei Provincial Clinical Medical Research Center for Nephropathy, Minda Hospital of Hubei Minzu University, Enshi, Hubei, China; 3 Department of Gastroenterology, Minda Hospital of Hubei Minzu University, Enshi, Hubei, China

**Keywords:** 1,8-cineole, hepatic fibrosis, HIF-1α/BNIP3, mitophagy, Pink1/Parkin

## Abstract

**Aim:**

The HIF-1α/BNIP3/mitophagy signaling pathway might be closely associated with hepatic fibrosis. Although 1,8-cineole (CIN) has been shown to improve fibrosis in several organs, direct evidence for its anti-hepatic fibrotic effects remains lacking. The objective of this study was to confirm the role of mitophagy in hepatic stellate cells (HSCs) and the role of the HIF-1α/BNIP3 signaling pathway in the pathogenesis of hepatic fibrosis. Additionally, *in vivo* and *in vitro* experiments were conducted to explore the effect of CIN on hepatic fibrosis.

**Methods:**

In a CCl_4_-induced hepatic fibrosis rat model and in CoCl_2_-treated HSCs, expression level of HIF-1α, BNIP3, α-SMA, collagen 1, PINK1, Parkin, p62 and LC3-II/LC3-I were examined, and the ubiquitinated Parkin level was detected following immunoprecipitation. In addition, the proliferation and apoptosis of HSCs were detected.

**Results:**

Our findings indicated that CIN significantly mitigated CCl_4_-induced hepatic fibrosis. The alleviation of hepatic fibrosis after CIN treatment was associated with a markedly decrease in HIF-1α, BNIP3, α-SMA, collagen 1, PINK1, Parkin and LC3-II/LC-I, as well as a signally increase in P62. Moreover, CIN, Mdivi-1 (mitophagy inhibitor) and Bafilomycin A1 (lysosomal inhibitors) significantly inhibited the proliferation of HSCs. The ubiquitinated Parkin level was significantly more prominent in the CoCl_2_ group, while the ubiquitinated Parkin level was observably decreased after CIN treatment. Additionally, CIN exhibited efficacy comparable to that of TRAIL (apoptosis-inducing agent) in significantly promoting apoptosis in hepatic stellate cells.

**Conclusion:**

CIN improves hepatic fibrosis by inhibiting HIF-1α/BNIP3/PINK1/Parkin-dependent mitophagy signaling and inducing apoptosis in activated HSCs.

## Graphical Abstract

**Figure:**
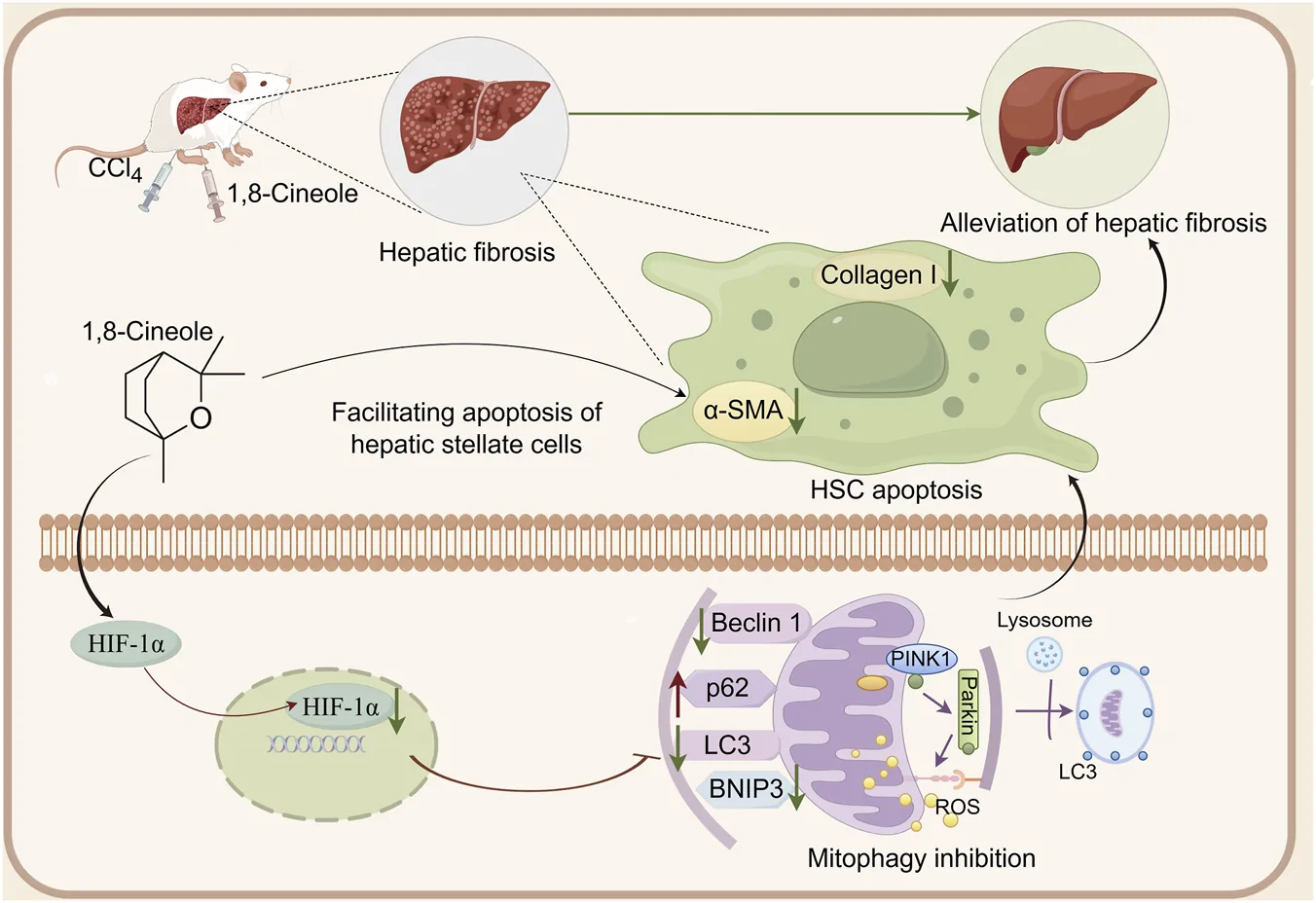

## 1 Introduction

Hepatic fibrosis is an inevitable stage in the progression of all chronic liver injuries to liver cirrhosis. It is characterized by the activation of the body’s damage repair mechanisms when the liver is repeatedly exposed to various chronic injuries. This leads to the formation of an extracellular matrix (ECM) mainly composed of collagen, along with excessive proliferation and deposition of the ECM in the liver. Among these processes, the excessive proliferation and reduced apoptosis of activated hepatic stellate cells (HSCs) are the central aspects of hepatic fibrosis. Hepatic fibrosis is the main pathological process underlying liver cirrhosis. Currently, cirrhosis has become one of the major diseases that seriously endanger human health and consume a substantial amount of public medical resources (Younossi et al., 2023). Statistics show that liver cirrhosis causes over 1.47 million deaths globally each year, and this figure is still increasing (Huang et al., 2023). At present, reversing liver cirrhosis remains a formidable challenge. However, blocking the hepatic fibrosis process before the development of liver cirrhosis can restore the liver to its normal state, which is currently the most effective approach for preventing and treating liver cirrhosis.

Hypoxia, as a key feature of the hepatic fibrosis microenvironment, can induce the expression of hypoxia-inducible factor 1(HIF-1α) (Hernández et al., 2020). HIF-1α can bind to the HRE in the promoter region of the Bcl-2/adenovirus E1B 19 kDa interacting protein 3 (BNIP3) gene, thereby upregulating the expression of BNIP3. The impact of BNIP3 on hepatic fibrosis may be achieved through the following mechanisms: On the one hand, BNIP3 can induce autophagy in HSCs (mainly mitochondrial autophagy), and this is mainly mediated through the PINK1/Parkin pathway. In the initial stage of HSCs activation, autophagy may support the proliferation and activation of HSCs by providing energy and nutrients, thus promoting the synthesis of ECM (Liu et al., 2020). On the other hand, overactivated BNIP3 may inhibit the apoptosis of HSCs, thereby promoting the development of hepatic fibrosis. In addition, the HIF-1α/BNIP3 pathway may also indirectly participate in the regulation of hepatic fibrosis by affecting processes such as inflammation and angiogenesis (Du et al., 2025; Michalak and Michalak, 2025; Bakleh and Al Haj Zen, 2025). Although the role of the HIF-1α/BNIP3 signaling pathway in chronic liver disease has been extensively explored, current research mainly focuses on the underlying basic mechanism. There is still a lack of exploration regarding clinical therapeutic strategies targeting this pathway. Natural products have demonstrated significant potential in regulating the HIF-1α/BNIP3 signaling pathway. However, more studies are required to verify their efficacy and safety.

1,8-Cineole (CIN) is a natural organic compound mainly present in the essential oils of Eucalyptus plants. The chemical name of CIN is 1,3,3-trimethyl-2-oxa-dicyclooctane. It has a molecular formula of C_10_H_18_O and a molecular weight of 154.25. CIN has a variety of biological activities and application values, and possesses pharmacological effects such as anti-inflammatory, antioxidant, anti-microbial, and liver-protecting effects (Yao et al., 2025). In our previous research, we found that natural products can inhibit the activation of HSCs by suppressing HSCs autophagy, thereby achieving the effect of alleviating hepatic fibrosis (Qin et al., 2023). Some studies have also shown that natural compounds derived from edible plants have anti-hepatic fibrosis effects and have few side effects, making them a promising source of drugs for the treatment of hepatic fibrosis (Xu et al., 2023). Therefore, in-depth research on the mechanism of action of CIN in hepatic fibrosis is helpful for the development of effective and low-toxic anti-hepatic fibrosis drugs, providing new strategies for solving the difficult problems of hepatic fibrosis treatment in clinical practice.

The objective of this study was to verify the role of the HIF-1α/BNIP3 signaling pathway in the pathogenesis of hepatic fibrosis and to explore the mechanism of CIN in anti-hepatic fibrosis through *in vivo* and *in vitro* experiments (Graphical Abstract). The aim was to further reveal the related mechanisms of hepatic fibrosis development and identify targets and drugs for reversing hepatic fibrosis.

## 2 Materials and methods

### 2.1 Animals and experimental protocols

In this study, forty 8-week-old male Sprague-Dawley rats, weighing 200–220 g, were purchased from the Laboratory Animal Centre of Hubei Minzu University (Enshi, China). The rats were housed in an environmentally controlled room maintained at 23 °C ± 3 °C and 55% ± 10% humidity, with a 12-h light cycle (06:00–18:00 light). Standard food and water were provided *ad libitum*. All animal experiments were conducted in accordance with the Guidelines for the Care and Use of Laboratory Animals of Hubei Minzu University, following the principle of minimizing animal suffering.

Sample size for the animal experiment was determined based on previous studies of hepatic fibrosis intervention using natural compounds in our laboratory and published literature. Based on a power analysis with an assumed effect size of 30% reduction in fibrosis area, a power of 0.80, and an alpha level of 0.05, a minimum of 8 animals per group was required. To account for potential attrition during the 8-week experimental period, 10 rats per group were included, consistent with our previous study.

After a one-week acclimation period, the rats were randomly divided into seven groups (n = 10/group) using a random number table: a control group, a model group, a CIN-L group, a CIN-H group, an PX-478 group, a mdivi-1 group, and a silymarin group. To induce hepatic fibrosis, all groups except the control group were intraperitoneally injected with 1.5 mL/kg body weight of CCl_4_/olive oil (2:3, v/v) every 3 days for 8 weeks. The control group received an equal volume of saline for 8 weeks. CCl_4_ was purchased from Macklin Biochemical Technology Co., Ltd. (Shanghai, China). The CIN-L group received 50 mg/kg of CIN orally once daily, while the CIN-H group received 200 mg/kg of CIN orally once daily. Silymarin, as a positive control drug for anti-hepatic fibrosis, was administered orally to the silymarin group at a dose of 50 mg/kg once daily. The model group received an equal volume of saline orally once daily, equivalent to the volume of CIN administered to the treatment groups. To verify whether CIN has the effect of inhibiting HIF-1α and suppressing mitophagy, we used HIF-1α inhibitor PX-478 and mitophagy inhibitor mdivi-1 in the experiment. CIN, silymarin, PX-478 and mdivi-1 were purchased from MedchemExpress (Shanghai, China). Rats in the HIF-1α inhibitor group received 100 mg/kg PX-478 via intraperitoneal injection once daily. Rats in the mitophagy inhibitor group were injected intraperitoneally with 50 mg/kg mdivi-1 once daily. All interventions lasted for 8 weeks. All histological analyses and quantitative measurements were performed by two independent researchers who were blinded to the group allocation of the samples. The code for group identity was revealed only after all data were collected and analyzed. After sacrificing the rats, the researchers collected liver tissues. Blood samples were collected for serum biochemical analysis (ALT, AST, TBIL), as shown in Table 1.

**TABLE 1:** Comparison of liver function levels in serum (mean ± SD).

Group	n	ALT	AST	TBIL
Control	10	65.28 ± 13.01	161.38 ± 23.46	10.58 ± 3.17
Model	10	168.67 ± 32.11^a^	269.53 ± 28.36^a^	43.87 ± 15.36^a^
CIN-L	10	118.47 ± 18.35^b^	220.72 ± 37.01^b^	30.84 ± 11.79^b^
CIN-H	10	83.92 ± 9.91^b^ ^,^ ^c^	186.87 ± 27.31^b^ ^,^ ^c^	18.35 ± 5.02^b^ ^,^ ^c^
PX-478	10	96.61 ± 11.71^b^	208.19 ± 25.11^b^	23.46 ± 8.42^b^
Silymarin	10	72.92 ± 10.72^b^	174.71 ± 25.62^b^	15.64 ± 5.43^b^

ALT, alanine aminotransferase; AST, aspartate aminotransferase; TBIL, total bilirubin.

^a^ *P* < 0.05 vs. control group.

^b^ *P* < 0.05 vs. model group.

^c^ *P* > 0.05 vs. Silymarin group.

### 2.2 Liver histopathology

A 4% paraformaldehyde solution was used as the fixative. After fixation in the 4% paraformaldehyde solution, the tissues were embedded in paraffin. Hematoxylin and eosin (HE) staining was carried out for histological structure analysis, and Masson’s trichrome staining was used for fibrosis area analysis. Each section was photographed using a light microscope (Olympus, Tokyo, Japan). Five random fields of each section were selected to assess the fibrotic area, which was evaluated using ImageJ 1.44 s software (National Institutes of Health, Bethesda, MD, United States). The percentage of the fibrotic area was calculated by determining the ratio of the collagen-stained area to the total area.

### 2.3 Immunohistochemistry and immunofluorescence

To detect the protein expression levels of HIF-1α, BNIP3, LC3, TOM20 and α-SMA in the liver tissues or cells of each group subsequently, immunohistochemistry and immunofluorescence were employed. First, the liver tissue sections were deparaffinized and hydrated, and then incubated with 5% H_2_O_2_. Next, heat-induced antigen retrieval was performed by transferring the slides into a sodium citrate buffer solution. The antibodies were diluted using an antibody diluent. The sections were blocked and then incubated overnight at 4 °C with rabbit anti-HIF-1α, BNIP3, and α-SMA antibodies (Abcam, Shanghai, China). For the negative control sections, PBS was used for incubation. The sections were incubated with fluorescence-labeled secondary antibodies for 1 h at 4 °C. Before staining the nucleus with DAPI and mounting the sections, the immunoreactivity was observed using a 3,3′-Diaminobenzidine tetrahydrochloride (DAB) solution. Finally, an optical microscope and the Image-Pro Plus 6.0 system (Media Cybernetics Inc., Bethesda, MD, United States) were used for analysis and semi-quantitative evaluation.

Quantification was performed on at least five random fields per sample at 200× magnification. The entire field of view was filled with tissue to ensure consistent background lighting for each photo during image acquisition. To convert green/red fluorescent monochrome photos into black-and-white images, Image-Pro Plus 6.0 software was used to select the same black level as the unified standard for judging the positivity of all images. Each image was analyzed to obtain the integrated optical density (IOD) and the pixel area (AREA). Then, the average optical density (AO) value was calculated (AO = IOD/AREA). A higher AO value indicates a higher expression level of the protein. Immunofluorescence was performed as described in the literature (Zoppolato et al., 2025).

### 2.4 Cell culture

The primary rat HSCs were obtained from the Cell Bank of the Chinese Academy of Sciences (Shanghai, China). These cells were incubated in a humidified 5% CO_2_ atmosphere at 37 °C and cultured in Dulbecco’s modified Eagle’s medium (DMEM) (Gibco, Waltham, MA, United States), supplemented with 10% fetal bovine serum (FBS) (Wisent, Nanjing, China). The cells were seeded in 12-well plastic plates at a density of 2 × 10^5^ cells per well. To activate primary HSCs, we used the activator PDGF-BB. The cells were induced for activation using 5 ng/mL PDGF-BB for 48 h. To study the changes of HSCs under hypoxic conditions,we used the chemical hypoxia inducer CoCl_2_. To verify whether CIN has an equivalent effect to the apoptosis inducer, we used the apoptosis inducer TRAIL. Mdivi-1 specifically inhibits Drp1, a key protein in mitochondrial fission, thereby blocking the initiation and recognition phases of PINK1/Parkin pathway-dependent mitophagy. Bafilomycin A1 (Baf A1), a lysosomal inhibitor, selectively targets vacuolar H^+^-ATPase (V-ATPase) to prevent lysosomal acidification, effectively impeding the fusion of autophagosomes with lysosomes and subsequent degradation processes. CoCl2, TRAIL, Mdivi-1, Baf A1 and PDGF-BB were purchased from MedchemExpress (Shanghai, China).

For the *in vitro* experiments, cells from the same batch were randomly assigned to different treatment groups using a random number generator. The HSCs were assigned to the following groups: the control group, the CoCl_2_ group, the CoCl_2_ + CIN-5 μM group, the CoCl_2_ + CIN-20 μM group, the CoCl_2_ + Mdivi-1 group, the CoCl_2_ + Baf A1 group, and the CoCl_2_ + TRAIL group. The control group received no treatment; the CoCl_2_ group was treated with CoCl_2_ alone; the CoCl_2_ + CIN-5 μM group and CoCl_2_ + CIN-20 μM group both received CoCl_2_ in combination with CIN; the CoCl_2_ + Mdivi-1 group was co-treated with CoCl_2_ and Mdivi-1; the CoCl_2_ + Baf A1 group received CoCl_2_ together with Baf A1; and the CoCl_2_ + TRAIL group was administered CoCl_2_ in combination with TRAIL. The concentrations of CoCl_2_ was set at 100 μM. The concentrations of CIN used in the CIN-5 μM and CIN-20 μM groups were 5 μM and 20 μM, respectively. The concentrations of mdivi-1 were 50 μM. The concentration of TRAIL was 1 μg/mL. The concentration of Baf A1 was 100 nM. The duration of cell intervention for each group was 24 h.

This study first used PDGF-BB as an activator of primary HSCs based on its well-established role as the most potent inducer of HSCs proliferation and survival during the early stages of hepatic fibrosis. For *in vitro* experiments, sample preparation and data collection were performed by one researcher, while data analysis was conducted by another researcher blinded to the treatment conditions.

*In vitro* experimental timeline: Primary HSCs were first activated using PDGF-BB for 48 h to induce proliferation. Subsequently, the activated HSCs were treated with the chemical hypoxia inducer CoCl_2_ (100 μM) either alone or in combination with various interventions (CIN, Mdivi-1, Baf A1, or TRAIL) for 24 h. After the 24-h intervention, cells were collected for subsequent assays including cell proliferation, RT-qPCR, ELISA, Western blot, immunoprecipitation, immune-fluorescence, and apoptosis detection, as described in Section 2.5, Section 2.6, Section 2.7, Section 2.8, Section 2.9, Section 2.10.

### 2.5 Cell proliferation assay

Cells were incubated for 24 h with various reagents at the indicated concentrations. According to the established protocol, cell proliferation was evaluated using a cell counting kit-8 (CCK-8) (Solarbio, Beijing, China). Cells were seeded in 96-well plates at a density of 1 × 10^4^ cells per well. After 24 h of treatment, 10 μL of CCK-8 solution was added to each well and incubated at 37 °C for an additional 2 h. The absorbance was measured at 450 nm using a microplate reader.

### 2.6 Reverse transcription and real-time quantitative polymerase chain reaction (RT-qPCR)

The mRNA expression of molecules was detected by RT-qPCR. Total RNA was extracted from rat liver tissues or cells using an RNA extraction kit. First, 1 μg of total RNA was used to synthesize first-strand cDNA. Subsequently, the cDNA sample was amplified in an ABI Prism 7500 Sequence Detection system (Applied Biosystems, Massachusetts, United States) for 40 cycles (95 °C for 3 s and 60 °C for 34 s) with specific oligonucleotide primers (Takara, Dalian, China). The target genes were relatively quantified using the 2^−ΔΔCT^ method, and the results were normalized to β-actin expression. Each sample was analyzed in triplicate.

Primer sequences for HIF-1α: Forward: 5′- TTG CTT TGA TGT GGA TAG CGA TA - 3′; Reverse: 5′-CAT ACT TGG AGG GCT TGG AGA AT - 3′. Primer sequences for BNIP3: Forward: 5′- GAA AAA CAG CAC TCT GTC TGA GGA A- 3′; Reverse: 5′-GAC CAA TCC CAT ATC CAA TCT GA - 3′. Primer sequences for β-actin: Forward: 5′- GTG ACG TTG ACA TCC GTA AAG A- 3′; Reverse: 5′-GTA ACA GTC CGC CTA GAA GCA C - 3′.

### 2.7 Enzyme linked immunosorbent assay (ELISA)

For cell culture experiments, after 48 h of cell culture, the supernatant and cell lysates were collected and diluted appropriately according to literature (Li et al., 2024) before detection. Each sample was measured in triplicate. The protein levels of HIF-1α, BNIP3, α-SMA, and collagen 1 were detected using an ELISA kit (Enzyme-linked Biotechnology, Shanghai, China) according to the manufacturer’s instructions. The optical density of the microplate was measured at 490 nm.

### 2.8 Western blot analysis

Total protein was extracted from liver tissue samples and cells using a standard protocol (Feng et al., 2025). Protein concentration was quantified via the BCA assay. Equal amounts of protein were separated by 15% sodium dodecyl sulfate-polyacrylamide gel electrophoresis (SDS-PAGE) and subsequently transferred to a polyvinylidene fluoride (PVDF) membrane. The membrane was blocked with 5% nonfat milk in TBST (0.1% Tween 20) for 1 h at room temperature. After a brief wash, the membrane was incubated overnight at 4 °C with the following primary antibodies diluted in TBST: rabbit monoclonal antibodies against PINK1 (1:1,000), Parkin (1:1,000), p62 (1:1,500), LC3-I (1:1,500),LC3-II(1:1,500), and GAPDH (1:2000). Subsequently, the membrane was incubated for 1 h at room temperature with horseradish peroxidase (HRP)-conjugated anti-rabbit secondary antibody (1:10,000). Following washes with TBST, protein bands were visualized using enhanced chemiluminescence (ECL). All samples were analyzed in triplicate. Densitometric analysis of all Western blot bands was performed using ImageJ software (National Institutes of Health, Bethesda, MD, United States). The grayscale value of each target protein band was first normalized to its corresponding internal control (GAPDH), and the results were expressed as fold change relative to the control group (set as 1.0). For the LC3-II/LC3-I ratio, the GAPDH-normalized value of LC3-II was divided by the GAPDH-normalized value of LC3-I. All results were expressed as fold change relative to the control group (set as 1.0). A minimum of three independent biological replicates were performed for each experiment, and each replicate was run in triplicate technical repeats.

### 2.9 Detection of Parkin ubiquitination levels following immunoprecipitation

To detect the ubiquitination level of Parkin, cells were first cultured to 80% confluence and lysed with RIPA lysis buffer (Beyotime) containing 1% protease inhibitor (Sigma) and 1% phosphatase inhibitor (Sigma). The cell lysates were incubated on ice for 30 min and then centrifuged at high speed (12,000×g, 4 °C, 10 min) to remove cell debris. The supernatants were collected and the protein concentration was determined using the BCA method (Thermo), and diluted to an appropriate concentration. Next, 1,000 μg of total protein was taken and incubated overnight with 20 μL of anti-Parkin antibody (Affinity) for immunoprecipitation. The antibody-protein mixture was incubated at 4 °C with rotation. The next day, 30 μL of Protein A/G magnetic beads (Thermo) were added and incubated for 2 h to pull down the immune complexes. After immunoprecipitation, the complexes were washed three times with lysis buffer for 5 min each. Then, the complexes were dissolved in SDS sample buffer containing *β*-mercaptoethanol and subjected to SDS-PAGE electrophoresis. After electrophoresis, the membrane was transferred to a PVDF membrane (Millipore), and the membrane was blocked with 5% non-fat milk (Beyotime) for 1 h. Subsequently, the membrane was incubated overnight with anti-ubiquitin antibody (Abcam) and anti-Parkin antibody (Affinity), respectively, and then incubated with HRP-labeled secondary antibody (Abcam) for 1 h. Finally, the membrane was developed with ECL chemiluminescent reagent (Thermo) and scanned using the ChemiDoc MP imaging system (Bio-Rad).

### 2.10 Apoptosis detection

Before washing the cell samples twice with PBS and centrifuging them at 1,000 × g for 5 min, we treated the HSCs with CoCl_2_, TRAIL, and different doses of CIN. Then, the cells were resuspended in cold D-Hanks buffer to a concentration of 1 × 10^6^ cells/ml. Next, 10 µL of Annexin V-FITC and 10 µL of propidium iodide (PI) were added to 100 µL of the cell suspension, and the mixture was incubated for 15 min at room temperature in the dark. Subsequently, to distinguish between living, early apoptotic, and necrotic cells, Annexin V-FITC binds to phosphatidylserine, which has flipped from the inner to the outer surface of the cell membrane, while propidium iodide can pass through the damaged cell membrane and bind to the DNA, emitting fluorescence under different laser excitations. Finally, the effects of CIN on the apoptosis of activated HSCs were evaluated via flow cytometry analysis.

Flow cytometry data were analyzed using CellQuest Pro software (BD Biosciences). To ensure consistency across all experimental groups, the same forward scatter (FSC)/side scatter (SSC) gate, established based on the control group, was applied uniformly to all samples. For the gating strategy, cells were first plotted on a FSC vs. SSC dot plot to identify the viable cell population based on size and granularity, excluding debris and cell clumps. Subsequently, Annexin V-FITC and PI fluorescence were detected using FL1 (530 nm) and FL2 (585 nm) channels, respectively. The quadrants were set based on unstained control cells and single-stained compensation controls. For the analysis of cell death: a. Annexin V-negative/PI-negative (lower left quadrant): viable cells. b. Annexin V-positive/PI-negative (lower right quadrant): early apoptotic cells. c. Annexin V-positive/PI-positive (upper right quadrant): late apoptotic/necrotic cells. d. Annexin V-negative/PI-positive (upper left quadrant): necrotic cells. The total apoptotic rate was calculated as the sum of the early apoptotic (Annexin V+/PI−) and late apoptotic (Annexin V+/PI+) populations.

### 2.11 Statistical analysis

All quantified data are presented as the mean ± standard deviation (SD). For comparisons between two groups, Student’s t-test was used. For comparisons among multiple groups, one-way analysis of variance (ANOVA) was performed, followed by Turkey’s post-hoc test for multiple comparisons. The statistical test used and the sample size (n) for each experiment are specified in the corresponding figure legends. A *P*-value less than 0.05 was considered statistically significant. All statistical tests were two-sided and analyzed using SPSS software version 24.0 (SPSS Inc., Chicago, IL, United States) or GraphPad Prism 9.0.

## 3 Results

### 3.1 CIN alleviates CCl_4_-induced hepatic fibrosis

**FIGURE 1:**
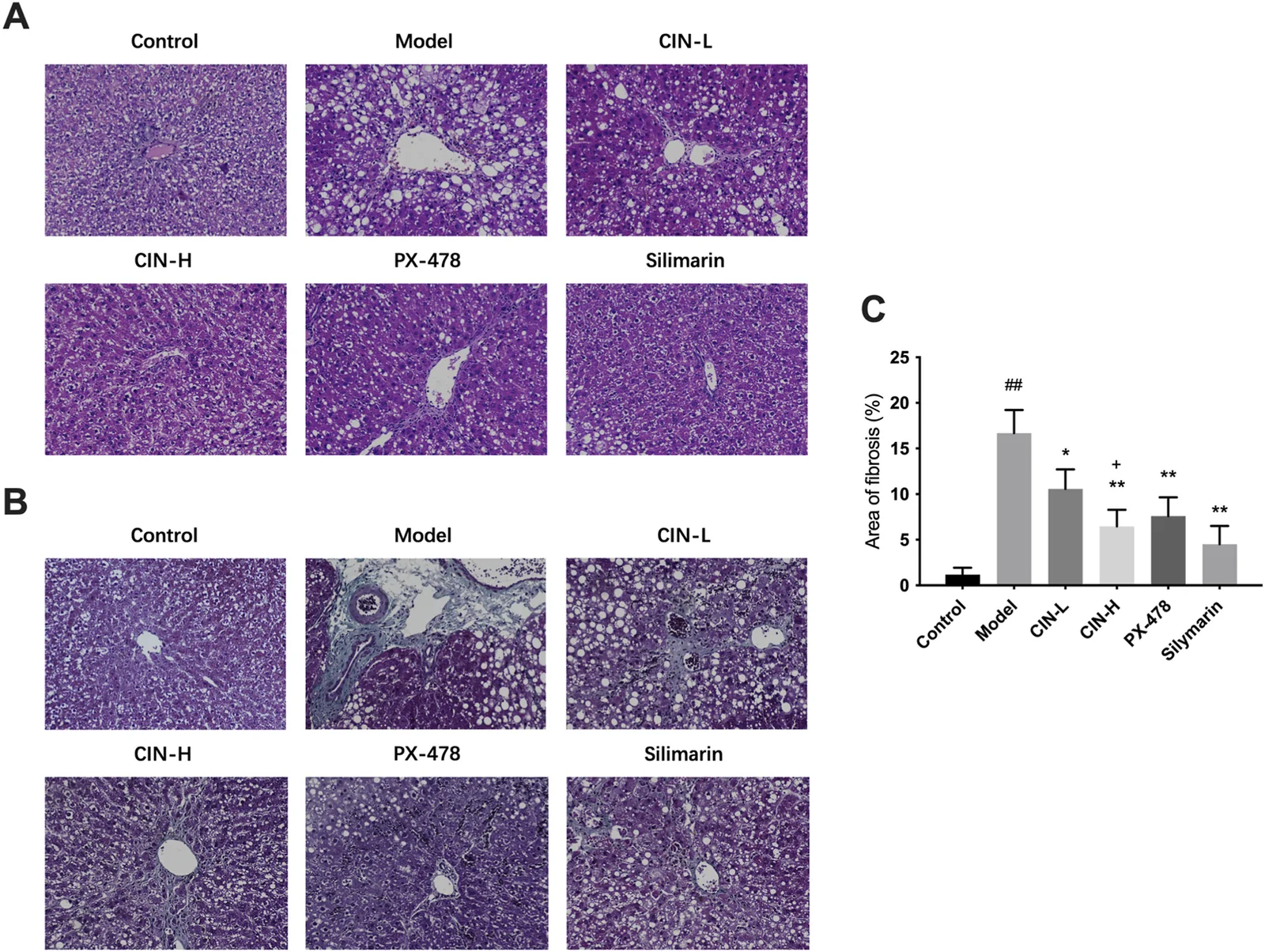
Histopathological change of liver tissue in each group. **(A)** Representative sections stained with HE are shown for each group (original magnification, 200×). **(B)** Representative sections stained with Masson’s trichrome are shown for each group (original magnification, 200×). **(C)** Quantitative analyses of hepatic fibrosis were conducted using Masson’s trichrome-stained sections. Results are expressed as the means ± SD (n = 10/group). CIN-L, low dose 1,8-cineole treated; CIN-H, high dose 1,8-cineole treated; PX-478, PX-478 (HIF-1α inhibitor) treated; Silymarin, silymarin treated. ^##^ *p* < 0.01 compared with the control group; ^*^ *p* < 0.05, ^**^ *p* < 0.01 compared with the model group; ^+^ *p* > 0.05 compared with the silymarin group.

Hematoxylin and eosin (HE) staining was performed to assess the level of liver damage (Figure 1A). In the control group, the liver tissue presented a normal lobular architecture, with hepatic cords radiating toward the central veins. In contrast, the model group showed fatty degeneration, along with ballooning changes and necrosis of hepatocytes. However, compared with the model group, the CIN, PX-478(HIF-1α inhibitor), and silymarin groups demonstrated significantly improved adipose degeneration of hepatocytes and reduced infiltration of inflammatory cells.

Masson’s trichrome staining was performed to assess the degree of hepatic fibrosis. The control group exhibited a normal liver architecture, while the model group showed extensive liver bridging fibrosis and significant collagen deposition. However, compared with the model group, the CIN, PX-478, and silymarin groups demonstrated a marked reduction in bridging fibrosis and collagen (*P* < 0.05). The high-dose CIN group and the silymarin group showed the same effect (*P* > 0.05) (Figure 1B).

Compared with the control group, the levels of ALT, AST and TBIL in the serum of the model group were significantly increased (*P* < 0.05). The levels of ALT, AST, TBIL in the CIN treatment groups, PX-478 and silymarin group were obviously improved compared with the model group (*P* < 0.05), and the changes showed a dose-dependent manner in the CIN treatment group. Compared CIN-H group with the silymarin group, there was no significant difference between the two groups (*P* > 0.05) (Table 1).

### 3.2 CIN inhibits the HIF-1α/BNIP3 signaling pathway *in vivo*

**FIGURE 2:**
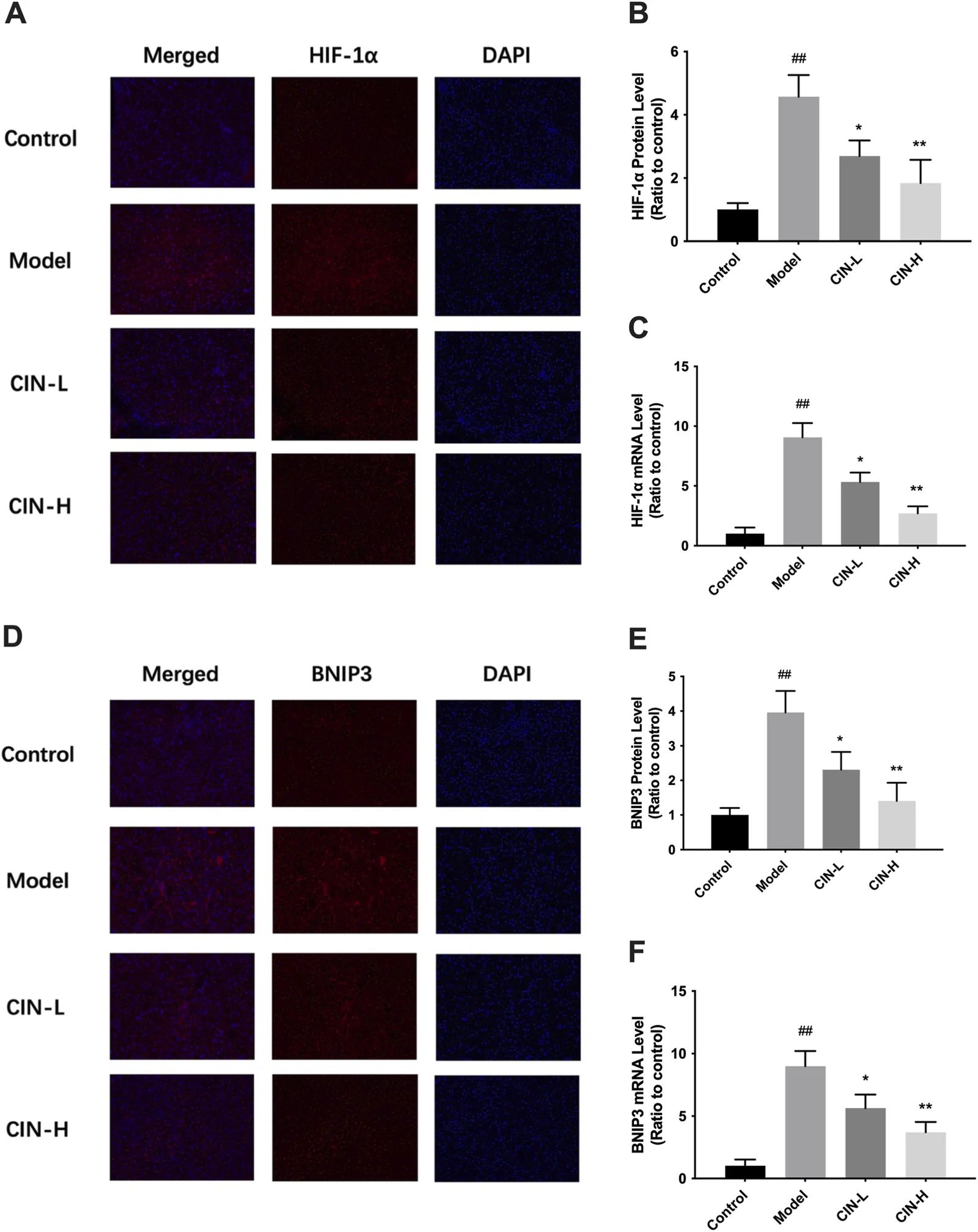
Effects of 1,8-cineole on HIF-1α and BNIP3 expression. **(A)** Immunofluorescence staining of HIF-1α (original magnification, 200×). **(B)** Quantification of HIF-1α staining by digital image analysis (n = 10). **(C)** The bar graph shows mean relative mRNA expression levels of HIF-1α in liver tissues (n = 10). Data are normalized to *β*-actin mRNA expression levels. **(D)** Immunofluorescence staining of BNIP3 (original magnification, 200×). **(E)** Quantification of BNIP3 staining by digital image analysis (n = 10). **(F)** The bar graph shows mean relative mRNA expression levels of BNIP3 in liver tissues (n = 10). Data are normalized to *β*-actin mRNA expression levels. Results are expressed as the means ± SD. CIN-L, low dose 1,8-cineole treated; CIN-H, high dose 1,8-cineole treated. ^##^ *p* < 0.01 compared with the control group; ^*^ *p* < 0.05, ^**^ *p* < 0.01 compared with the model group.

Compared with the control group, the mRNA and protein levels of HIF-1α and BNIP3 were significantly elevated in the model group (P < 0.01). However, in the CIN group, the expression levels of HIF-1α and BNIP3 were markedly decreased in a dose-dependent manner. These findings suggest that the HIF-1α/BNIP3 pathway is involved in the pathogenesis of hepatic fibrosis, and CIN effectively inhibits the HIF-1α/BNIP3 pathway to alleviate hepatic fibrosis (Figure 2).

### 3.3 CIN suppresses activity of HSCs *in vivo*

**FIGURE 3:**
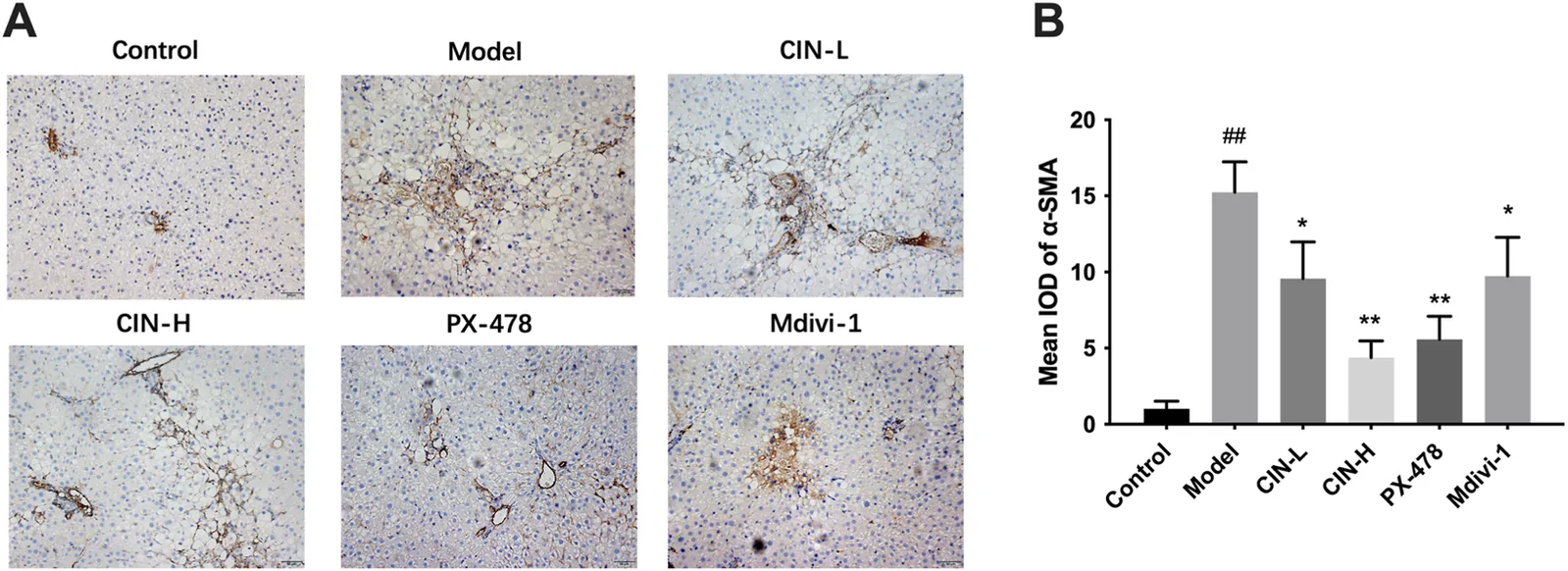
Effects of 1,8-cineole on α-SMA expression and hepatic distribution. **(A)** Immunohistochemical staining of α-SMA (original magnification, 200×). **(B)** Quantification of α-SMA staining by digital image analysis (n = 10). The ocular fields per specimen were assessed as mean IOD, and data are presented as mean ± SD. CIN-L, low dose 1,8-cineole treated. CIN-H, high dose 1,8-cineole treated. PX-478, PX-478(HIF-1α inhibitor) treated. Mdivi-1, mdivi-1 (Mitophagy inhibitor) treated. ^##^ *p* < 0.01 compared with the control group;^*^ *p* < 0.05, ^**^ *p* < 0.01 compared with the model group.

α-SMA serves as a crucial molecular marker for the activation of HSCs during hepatic fibrosis. The expression of α-SMA was examined by immunohistochemical staining. When compared with the control group, the model group showed a significant increase in α-SMA-positive cells within the fibrotic areas and vessel walls of liver sections (*P* < 0.01). In contrast, the CIN treatment groups, along with the PX-478 and Mdivi-1 (Mitophagy Inhibitor) groups, had fewer α-SMA-positive cells compared to the model group (*P* < 0.05). Moreover, the expression level was inversely proportional to the dosage of CIN (Figure 3). Semiquantitative analysis using Image-Pro Plus 6.0 confirmed the findings from immunohistochemistry. These data indicate that HIF-1α regulates the activation of HSCs, and CIN inhibits the activation of HSCs in fibrosis.

### 3.4 CIN inhibits the HIF-1α/BNIP3 signaling pathway *in vitro*

**FIGURE 4:**
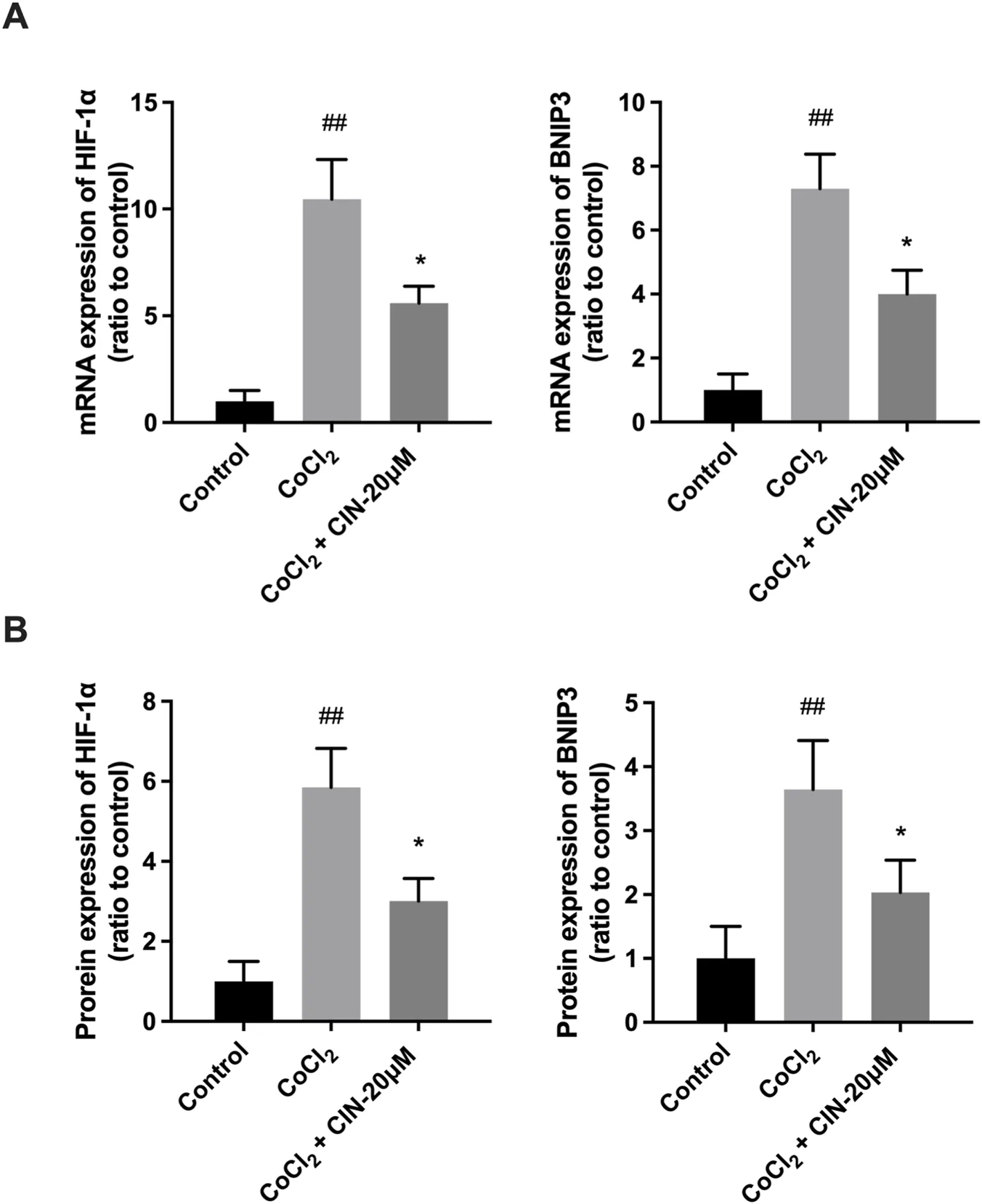
Effect of 1,8-cineole on the mRNA and protein expression of HIF-1α/BNIP3 signaling pathway in HSCs. **(A)** The bar graph shows mean relative mRNA expression levels of HIF-1α and BNIP3 in HSCs (n = 3). Data are normalized to *β*-actin mRNA expression levels. **(B)** The bar graph represents mean protein expression levels of HIF-1α and BNIP3 (n = 3). Results are expressed as the means ± SD. CoCl_2_, CoCl_2_ treated; CIN- 20 μM, 20 μM 1,8-cineole treated. ^#^ *p* < 0.05, ^##^ *p* < 0.01 compared with the control group; ^*^ *p* < 0.05 compared with the CoCl_2_ group.

RT-qPCR and ELISA results indicated that, compared with the control group, the expression levels of HIF-1α and BNIP3 increased significantly in the CoCl_2_ group. Conversely, CIN significantly decreased the production of HIF-1α and BNIP3 stimulated by CoCl_2_ (*P* < 0.05) (Figure 4). These findings suggest that the HIF-1α/BNIP3 signaling pathway plays a crucial role in hepatic fibrosis, and CIN inhibits this signaling pathway.

### 3.5 CIN suppresses activity of HSCs *in vitro*

**FIGURE 5:**
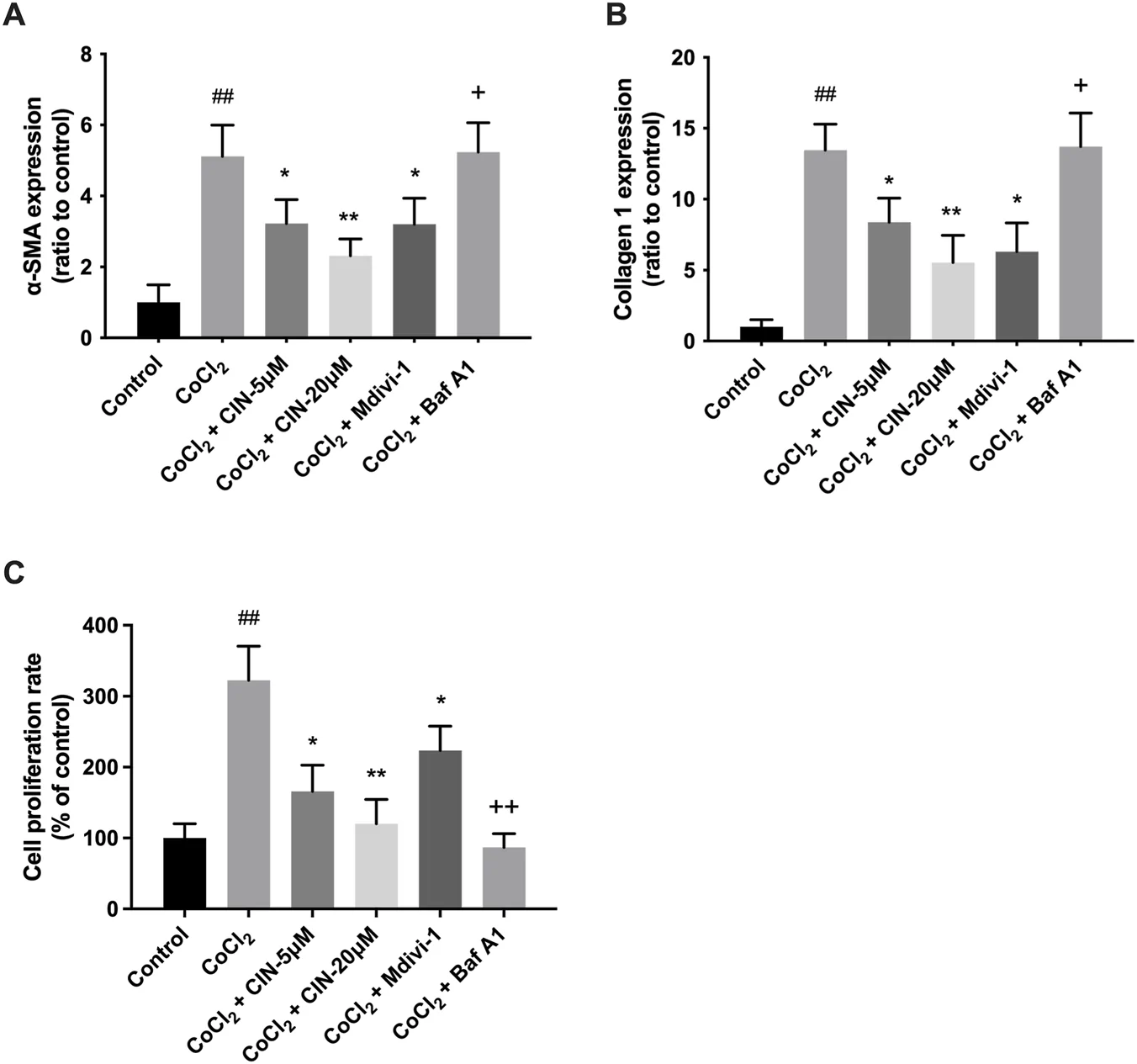
Effect of 1,8-cineole on activity of HSCs *in vitro*. **(A)** ELISA analysis of α-SMA protein expression levels (n = 3). **(B)** ELISA analysis of collagen 1 protein expression levels (n = 3). **(C)** Cell proliferation analysis of proliferation rate of HSCs (n = 3). Results are expressed as the means ± SD. CoCl_2_, CoCl_2_ treated. CIN-5 μM, 5 μM 1,8-cineole treated. CIN-20 μM, 20 μM 1,8-cineole treated. Mdivi-1, mdivi-1 (Mitophagy inhibitor) treated. Baf A1, Bafilomycin A1 (lysosomal inhibitors) treated. ^##^ *P* < 0.01 compared with the control group, ^*^ *p* < 0.05, ^**^ *p* < 0.01 compared with the CoCl_2_ group, ^+^ *p* < 0.05, ^++^ *p* < 0.01 compared with the Mdivi-1 group.

ELISA results indicated that the levels of α-SMA and collagen 1 were significantly elevated in the CoCl_2_ group compared with the control group. However, the CoCl_2_-induced upregulation of α-SMA and collagen 1 was dose-dependently inhibited by CIN. Additionally, the CoCl_2_-induced upregulation of α-SMA and collagen 1 was suppressed by Mdivi-1 (*P* < 0.05),but group Baf A1 showed an opposite effect to that of the Mdivi-1 group (*P* < 0.05) (Figures 5A,B). These findings suggest that the activation of HSCs is regulated by the HIF-1α/BNIP3 pathway and mitophagy, and CIN inhibits the activation of HSCs.

As depicted in Figure 5C, the proliferation rate of HSCs in the CoCl_2_ group increased significantly compared with the control group (*P* < 0.01). Conversely, CIN decreased cell proliferation in a dose-dependent manner (*P* < 0.05). Additionally, Mdivi-1 and Baf A1 remarkably reduced cell proliferation (*P* < 0.05),Moreover, group Baf A1 more potently inhibited cell proliferation compared with the Mdivi-1 group. These findings confirm that the proliferation of HSCs is regulated by the HIF-1α/BNIP3 pathway and mitophagy, and CIN effectively inhibits the proliferation of HSCs.

### 3.6 CIN inhibits mitophagy in HSCs

**FIGURE 6:**
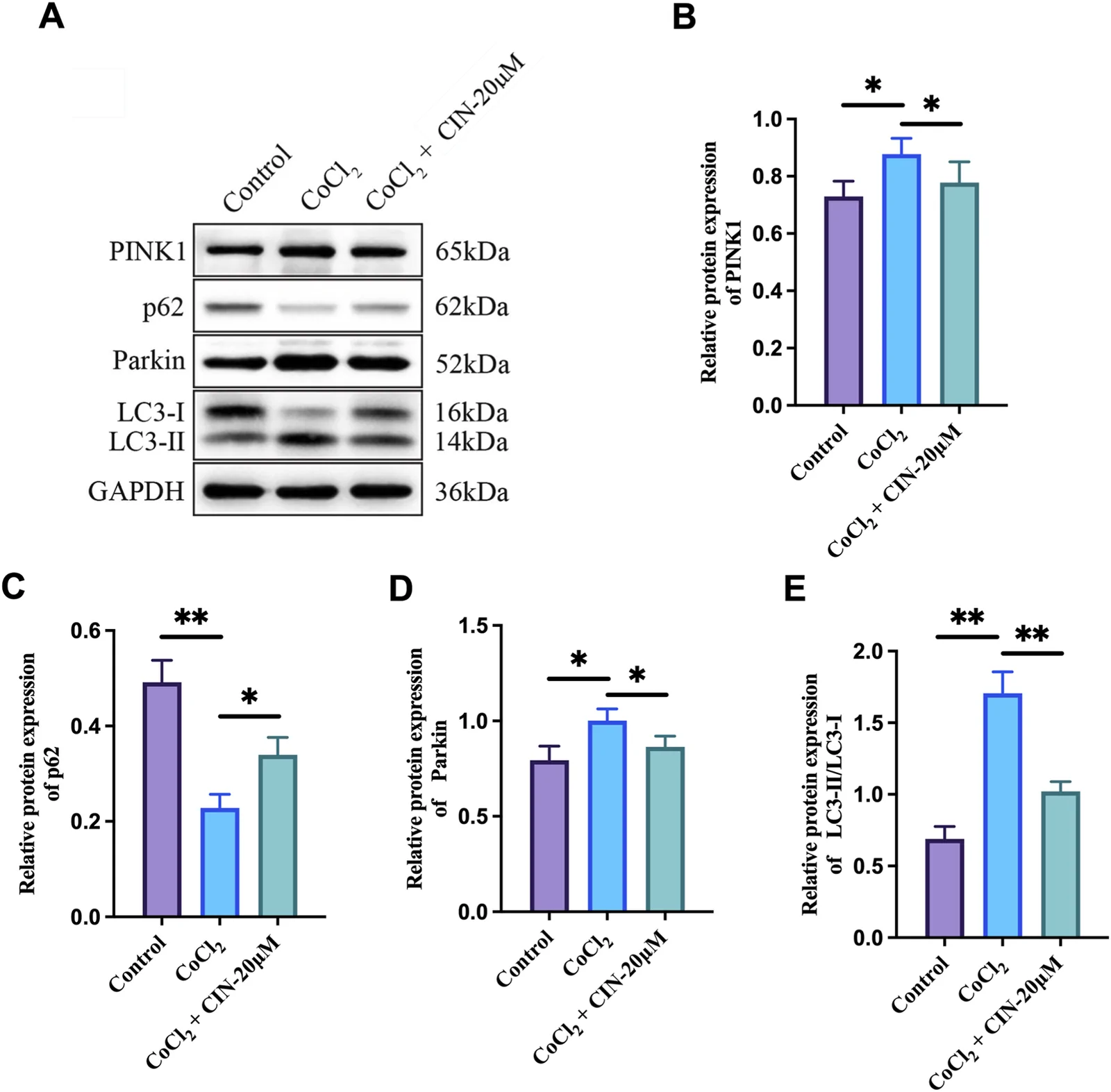
Effect of 1,8-cineole on the mitophagy in HSCs. **(A)** Representative western blot analysis of PINK1, P62, Parkin and LC3-II/LC3-I protein expression levels in HSCs. **(B)** The bar graph represents mean protein expression levels of PINK1 (n = 3). **(C)** The bar graph represents mean protein expression levels of P62 (n = 3). **(D)** The bar graph represents mean protein expression levels of Parkin (n = 3). **(E)** The bar graph represents mean protein expression levels of LC3-II/LC3-I (n = 3). Quantitative analysis of the LC3-II/LC3-I ratio. The grayscale value of each LC3 band was first normalized to the corresponding GAPDH loading control before calculating the ratio. Results are expressed as the means ± SD. CoCl_2_, CoCl_2_ treated; CIN-20 μM, 20 μM 1,8-cineole treated. ^*^ *p* < 0.05, ^**^ *p* < 0.01 compared with the HIF-1α or control group.

Western blot analysis (Figure 6A) indicated that the protein expression level of PINK1 was significantly higher in the CoCl_2_ group than in the control group. In contrast, the CoCl_2_+CIN-20 μM group showed a notable decrease compared to the CoCl_2_ group (*P* < 0.05) (Figure 6B). The protein expression of p62 was significantly reduced in the CoCl_2_ group (*P* < 0.05), while it was significantly increased in the CoCl_2_ +CIN-20 μM group (*P* < 0.05) (Figure 6C). The Parkin protein expression was significantly elevated in the CoCl_2_ group (*P* < 0.05), and this increase was inhibited in the CoCl_2_ +CIN-20 μM group (*P* < 0.05) (Figure 6D). After normalization to the GAPDH loading control, the LC3-II/LC3-I ratio was significantly upregulated in the CoCl_2_ group compared with the control group (*P* < 0.05), and this upregulation was markedly suppressed in the CoCl_2_ + CIN-20 μM group (*P* < 0.05) (Figure 6E).

**FIGURE 7:**
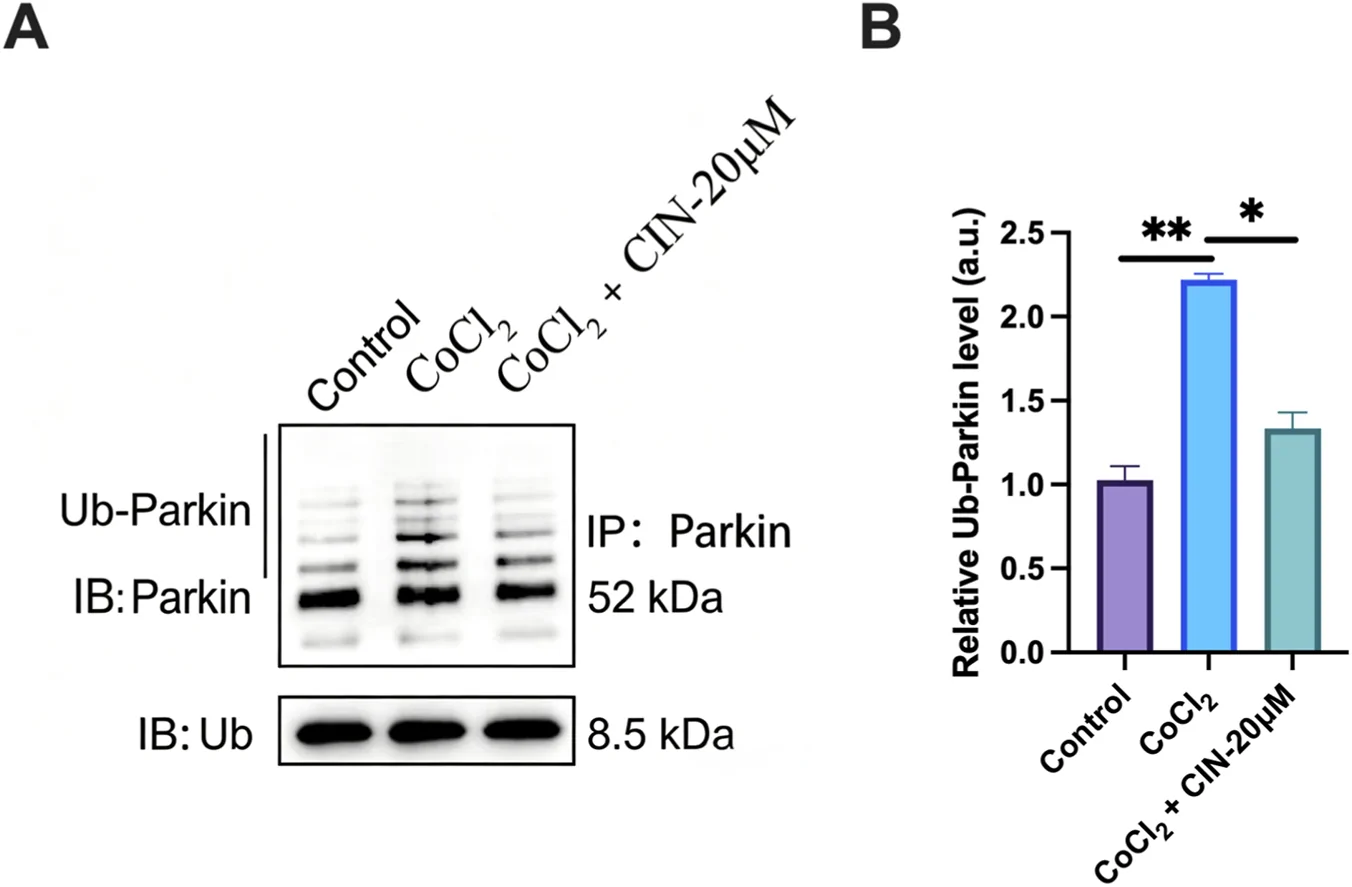
Effect of 1,8-cineole on the expression of Parkin ubiquitination in HSCs. **(A)** Representative western blot analysis of Parkin ubiquitination in HSCs. IP: Parkin; IB: Parkin; IB: Ub. **(B)** The bar graph represents mean ubiquitin Parkin (Ub-Parkin) level in different groups (n = 3). Results are expressed as the means ± SD. Quantitative analysis of Parkin ubiquitination was performed on the high molecular weight smear region (above −55 kDa) of the ubiquitin immunoblot. The −52 kDa bands corresponding to free/unmodified Parkin were excluded from the analysis. CoCl_2_, CoCl_2_ treated; CIN-20 μM, 20 μM 1,8-cineole treated. ^*^ *p* < 0.05, ^**^ *p* < 0.01 compared with the CoCl_2_ or control group.

The levels of ubiquitinated Parkin (Ub-Parkin) and free ubiquitin (Ub) were detected by immunoprecipitation (IP) and Western blot. Results showed that CoCl_2_ treatment significantly enhanced ubiquitination of Parkin as indicated by increased dispersion of Ub-Parkin bands (relative level in quantitative plot was significantly higher, *P* < 0.01) compared with the control group. Co-treatment with CIN-20 μM resulted in a significant reduction in Parkin ubiquitination level compared with CoCl_2_ treatment alone (*P* < 0.05), but still higher than the control group. Free ubiquitin (Ub) levels were not significantly different among the groups (Figure 7). Quantification of ubiquitinated Parkin was performed exclusively on the high molecular weight ubiquitin signals (above 55 kDa), excluding the free Parkin bands at approximately 52 kDa.

The above results suggest that the HIF-1α/BNIP3 signaling pathway can induce PINK1/Parkin-mediated mitophagy, and CIN can inhibit mitophagy in HSCs.

### 3.7 CIN induces apoptosis of HSCs

**FIGURE 8:**
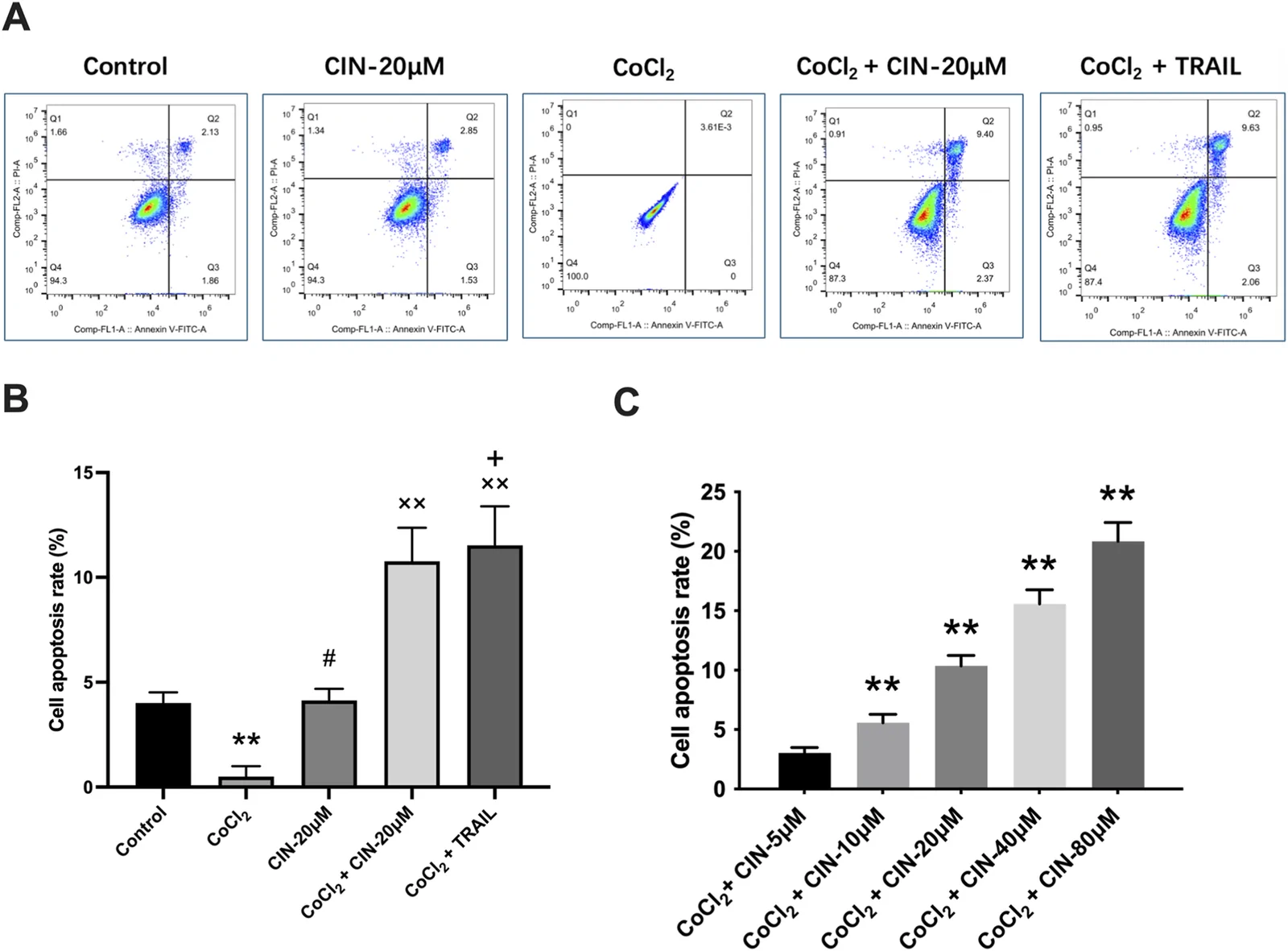
Effect of 1,8-cineole on apoptosis rate of HSCs. **(A)** Representative pictures of the apoptosis of HSCs in each group detected by flow cytometry. **(B)** Quantification of HSCs apoptosis in each group (n = 3). **(C)** Effects of 1,8-cineole at different doses on apoptosis rate of HSCs (n = 3). Results are expressed as the means ± SD. CoCl_2_, CoCl_2_ treated; TRAIL, TRAIL (apoptosis inducer) treated. CIN-5 μM, 5 μM 1,8-cineole treated; CIN-10 μM, 10 μM 1,8-cineole treated; CIN-20 μM, 20 μM 1,8-cineole treated; CIN-40 μM, 40 μM 1,8-cineole treated; CIN-80 μM, 80 μM 1,8-cineole treated. ^**^ *p* < 0.01 compared with the control group. ^#^ *p* > 0.05 compared with the control group. ^××^ *p* < 0.01 compared with the CoCl_2_ group. ^+^ *p* > 0.05 compared with the CoCl_2_ + CIN-20 μm group.

Compared with the control group, the apoptosis rate of HSCs in the CoCl_2_ group was significantly decreased (*P* < 0.01), but there was no significant change in the apoptosis rate of HSCs in the CIN-20 μm group (*P* > 0.05). In contrast, CIN and TRAIL (apoptosis inducer) increased the cell apoptotic percentage markedly compared with the CoCl_2_ group (*P* < 0.01). Moreover, CIN and TRAIL induced apoptosis of HSCs with similar effects (*P >* 0.05). Additionally, CIN increased the cell apoptotic percentage significantly in a dose-dependent manner (*P* < 0.05) (Figure 8). These results suggest that hypoxia may inhibit the apoptosis of HSCs, while CIN may induce the apoptosis of HSCs. Hypoxia induced by CoCl_2_ may be a necessary condition for CIN-induced apoptosis of HSCs.

## 4 Discussion

Edible plant-derived natural compounds have few side effects in the treatment of hepatic fibrosis and are a promising source of drugs for treating hepatic fibrosis (Xu et al., 2023). Previous studies (Qin et al., 2023) by our research group have found that natural products can inhibit the activation of HSCs by suppressing HSCs autophagy, achieving the effect of alleviating hepatic fibrosis. Globally, numerous studies on CIN have obtained favorable outcomes. Some studies have found that eucalyptus oil (with CIN as the main component) can reduce the deposition of ECM, relieve oxidative stress in renal tissue, and reduce the inflammatory response through the TGF-β1/Smad3 signaling pathway, thus exerting an anti-renal fibrosis effect (Li et al., 2025). CIN can alleviate bleomycin-induced pulmonary fibrosis by regulating M2 macrophage polarization (Rui et al., 2022). Therefore, CIN may be a potential therapeutic drug for idiopathic pulmonary fibrosis. CIN can reduce cardiac fibrosis in diabetic rats and inhibit the activation of high glucose-induced cardiac fibrosis by regulating the GRK5/NFAT signaling pathway (Wang et al., 2023). Therefore, CIN has been proven to alleviate fibrosis in multiple organs. Abdollahi et al. (Abdollahi et al., 2024) found that CIN can play a protective role in the liver by inhibiting the NF-κB signaling pathway to regulate oxidative stress and the inflammatory response. A study (Barbhuiya et al., 2025) from India showed that the cooking spice extract CIN has a significant therapeutic effect on metabolic dysfunction-associated steatotic liver disease. Zhang et al. (2024) used a biomarker-driven research method based on multi-omics and bioinformatics and found that Eucommia ulmoides leaves (with CIN as the main active ingredient) may protect the cirrhotic liver from excessive energy consumption and the activation of HSCs by inhibiting the oxidative phosphorylation pathway. By searching multiple databases, we found that there is currently a lack of research on the direct effect of the CIN monomer on hepatic fibrosis. This study demonstrates for the first time that CIN exerts a significant anti-hepatic fibrotic effect. The therapeutic effect was found to be proportional to the dosage of CIN, with the high-dose group showing an effect comparable to that of the positive control drug silymarin (Figure 1; Table 1).

Studies (Liu et al., 2020) have confirmed that the expression of BNIP3 is significantly increased in hypoxic-treated HSCs and in a mouse model of schistosomal hepatic fibrosis. After using the HIF-1α inhibitor YC-1, the expression of BNIP3 is inhibited. Shi et al. (2023) confirmed through *in vivo* and *in vitro* experiments that during the treatment with the anti-tumor drug irinotecan, liver injury can be induced via the HIF-1α/BNIP3 pathway, and inhibiting HIF-1α can effectively reverse this liver injury. It can be seen that the HIF-1α/BNIP3 pathway positively regulates activity of mitophagy by upregulating the expression of BNIP3 during the process of hepatic fibrosis and is involved in the pathophysiological process of hepatic fibrosis. Specifically, mitophagy is primarily mediated through the PINK1/Parkin pathway (Narendra et al., 2024). Under physiological conditions, PINK1 protein expression remains low. However, mitochondrial damage leads to a reduction or complete loss of mitochondrial membrane potential, triggering the accumulation of PINK1 on the outer mitochondrial membrane. There, PINK1 recruits and phosphorylates the E3 ubiquitin ligase Parkin, activating its function. Subsequently, Parkin promotes ubiquitination of mitochondrial proteins, while adaptor proteins facilitate the linkage between ubiquitin and LC3, thereby tethering mitochondria to autophagosomes and forming mitophagosomes. Ultimately, these mitophagosomes fuse with lysosomes, leading to mitochondrial degradation (Narendra et al., 2024).

Mechanistically, we discovered that CIN suppresses the HIF-1α/BNIP3/PINK1/Parkin signaling pathway to inhibit mitophagy in activated HSCs. This conclusion is supported by multiple lines of evidence: (1) CIN dose-dependently reduced the mRNA and protein levels of HIF-1α and BNIP3 both *in vivo* and *in vitro* (this refers to Figures 2, 4); (2) It decreased the expression of mitophagy markers PINK1, Parkin, and the LC3-II/LC3-I ratio, while increasing p62 levels (this refers to Figure 6); (3) It significantly reduced the hypoxia-induced ubiquitination of Parkin, a key step in PINK1/Parkin-mediated mitophagy (this refers to Figure 7). These results suggest that the anti-fibrotic effect of CIN is closely linked to its ability to suppress the protective mitophagy that sustains activated HSCs.

A particularly notable finding from this study is the differential effect observed between the mitophagy inhibitor Mdivi-1 and the lysosomal inhibitor Baf A1. Mdivi-1 significantly suppressed HSC activation, whereas Baf A1 exhibited no such inhibitory effect (this refers to Figures 5A,B). This discrepancy can be explained by their different mechanisms of action. Mdivi-1 specifically blocks the initiation of mitophagy, thereby removing the protective mechanism for HSCs. In contrast, Baf A1 completely blocks the final degradative stage of autophagy, leading to an accumulation of autophagosomes. This complete blockade likely constitutes a substantial cellular stress, potentially inducing widespread cell death rather than merely inhibiting HSC activation. Therefore, the targeted and early inhibition of mitophagy, as achieved by Mdivi-1 and CIN, appears to be a more effective strategy for suppressing HSC survival and activity. Future studies could explore the use of more specific BNIP3 inhibitors to further validate this pathway as a therapeutic target.

In activated HSCs under hypoxic conditions, the main function of BNIP3 is not to directly induce apoptosis but rather to potently induce protective autophagy, especially mitophagy. BNIP3 disrupts the binding between the anti-apoptotic protein BCL-2 and the autophagy key protein Beclin 1, thereby activating the autophagic process. BNIP3 is localized on the outer mitochondrial membrane and can recruit autophagosomes, selectively clearing mitochondria with impaired function due to hypoxia and oxidative stress. Through the above mechanisms (especially blocking the mitochondrial apoptotic pathway and reducing oxidative stress), coupled with the possible inhibitory effect of high-level autophagy itself on the apoptotic signaling pathway, the autophagy activated by BNIP3 overall inhibits the apoptosis of HSCs. This enables activated HSCs to continuously survive and proliferate in a harsh hypoxic microenvironment and secrete a large amount of ECM, thus promoting the development of hepatic fibrosis (Huang et al., 2025). Inhibiting the HIF-1α/BNIP3/mitophagy axis can significantly reduce the degree of hepatic fibrosis in animal models, which is directly related to the increased apoptosis of HSCs (Shi et al., 2023). Through this study, we discovered for the first time that CIN could promote the apoptosis of HSCs, and hypoxia may be a necessary condition for the apoptosis of HSCs induced by CIN. We speculate that hypoxia is the upstream driving force for the activation of this protective pathway and that CIN acts to block this defense mechanism rather than directly initiate apoptosis.

Our previous study (Qin et al., 2023) found that natural products could inhibit HSCs activation by suppressing general autophagy. The current study advances this knowledge by pinpointing a specific form of autophagy—mitophagy—and the specific signaling cascade involved. The hypoxia-driven HIF-1α/BNIP3 pathway is presented as a key trigger for this process, and CIN as a promising natural inhibitor of this entire axis. This shift from a general observation to a specific molecular target is a critical step for developing more effective anti-fibrotic therapies.

This study has several limitations that warrant consideration. Firstly, while we demonstrate a strong association between the inhibition of the HIF-1α/BNIP3 pathway and the induction of HSC apoptosis, we did not perform direct gain- or loss-of-function experiments (e.g., overexpressing HIF-1α or BNIP3) to establish a definitive causal relationship (Figures 2, 4). Future studies using such approaches are necessary to confirm the causal link. Secondly, a potential technical limitation of this study is that CoCl_2_ has been reported to act as a fluorescence quencher in certain experimental conditions. Although we performed multiple wash steps with PBS prior to flow cytometry analysis to minimize residual CoCl_2_ in the culture medium, we cannot completely exclude the possibility that intracellular Co^2+^ might have a minor quenching effect on Annexin V-FITC or PI fluorescence. However, the observed pro-apoptotic effect of CIN was dose-dependent (Figure 8C) and was comparable to that of the apoptosis inducer TRAIL (Figure 8B), which supports the validity of our conclusions. Thirdly, the findings are based on a single animal model (CCl_4_-induced) and an *in vitro* chemical hypoxia model (CoCl_2_). Validation using other models of hepatic fibrosis, such as bile duct ligation (BDL) or thioacetamide (TAA) models, would strengthen the generalizability of the conclusions.

In summary, our findings demonstrate that CIN inhibits mitophagy and induces apoptosis of activated HSCs. These effects are strongly associated with the suppression of the HIF-1α/BNIP3/PINK1/Parkin signaling pathway, suggesting a potential mechanism by which CIN ameliorates hepatic fibrosis. This study provides a foundation for future research aimed at validating this pathway as a therapeutic target and developing CIN as a potential treatment for hepatic fibrosis.

## Data Availability

The original contributions presented in the study are included in the article/supplementary material, further inquiries can be directed to the corresponding author.
